# An Evaluation of Non-Uniform Grade Distribution with the Emergency Medicine Off-Service Standardized Letters of Evaluation

**DOI:** 10.30476/JAMP.2022.93990.1561

**Published:** 2022-07-01

**Authors:** JORDAN GOWMAN, BERNADETTE DAZZO, JACE COON, TRACY KOEHLER, RYAN OFFMAN, JOSEPH BETCHER

**Affiliations:** 1 West Michigan Emergency Medicine Residency, Trinity Health West Michigan, Michigan State University, Michigan, USA; 2 Mercy Health Muskegon, Michigan State University, Michigan, USA

**Keywords:** Emergency medicine, Residency, Evaluation

## Abstract

**Introduction:**

Standardized Letters of Evaluation (SLOEs) are designed to objectively compare medical students to their peers for completed emergency medicine (EM) rotations to be used in the EM residency match. In an attempt to adapt quickly to the lack of availability of in-person EM rotations due to COVID restrictions, “off-service” SLOEs (OSLOEs) were allowed in place of traditional SLOEs. The purpose of this study was to assess the utility of OSLOEs for candidate selection during the 2020-21 application cycle at a single EM residency.

**Methods:**

A retrospective cohort review of all OSLOEs submitted during the 2020-21 academic year to an EM residency program was performed. A total of 270 OSLOES were eligible for review. Summary statistics were calculated for the study variables recorded, including global rank, grade, categorical details, and rank.

**Results:**

Of the 270 OSLOEs reviewed, 61.9% ranked candidates in the top 10% of their class, with 95% being ranked in the top two categories. Over 90% of students were graded as honors or high pass and over 75% of students were ranked in the top 1/3 for each specific OSLOE category.

**Conclusion:**

Our findings reveal questionable utility of the objective measures in the OSLOE as there are signs it may suffer from non-uniform grade distribution, leading to low utility for candidate selection. Our data shows marked over-ranking within the highest 2 categories. EM program directors and faculty should use caution as the OSLOE may not carry the same weight as a traditional SLOE when objectively evaluating prospective students for a match into EM.

## Introduction

Emergency Medicine (EM) residency programs first started using standardized letters of recommendations in place of traditional narrative letters of recommendations (NLORs) for their medical school applicants in 1995 ( 1 ). NLORs were largely subjective and difficult to interpret. This created a need to increase objectivity, as well as provide a way to evaluate an applicant’s interpersonal and clinical skills ( 2 - 4 ). Standard letters of recommendation have gone through several iterations, with the most recent version being the standardized letter of evaluation (SLOE) developed by the Council of Residency Directors in Emergency Medicine (CORD) in 2014 ( 5 ). SLOEs are completed by EM physicians or a committee of EM physicians and use ranking for specific categories, along with narrative feedback to combine objective and subjective measures of a candidate. A survey of EM directors in 2018 showed that SLOEs have developed a crucial role in the application process with 80% of directors requiring at least one SLOE to offer an interview and 38% requiring at least two SLOEs. Furthermore, most directors ranked SLOEs among the top considerations for ranking an applicant ( 4 , 6 , 7 ) .

  During the COVID-19 pandemic, the Association of American Medical Colleges issued various non-clinical mandates and individual universities and hospitals adopted policies for their own students’ safety ( 8 - 10 ). With safety in mind, the EM community encouraged students to only participate in home EM rotations, decreasing students’ ability to obtain multiple SLOEs or even a single SLOE if no EM rotations were available ( 11 ). To compensate, CORD implemented “off-service” or “other rotation” SLOEs (OSLOEs), which could be completed by non-EM attendings. This option provided additional opportunities for students to receive both narrative feedback and objective rankings on an off-service, or non-EM clinical rotation, to be included in their application to the EM match. This letter allows for details about the student’s performance during an off-service rotation, including data regarding grades distributed, categorical clinical rankings among student peers, global assessment ranking, as well as narrative comments regarding the student.

 With the addition of non-EM physicians contributing to standardized letters of evaluation for EM residency applicants, there is concern related to the utility of OSLOEs as a substitute for a SLOE. The purpose of this study was to assess the utility of OSLOEs for candidate selection for a single EM residency during the 2020-21 application cycle. 

## Methods

A retrospective cohort study utilizing data from OSLOEs submitted during the 2020-21 academic year to an EM residency program was performed. Students’ applications were downloaded from the Electronic Residency Application Service (ERAS) following submission to the residency program. OSLOEs submitted within the applications were de-identified and data points from these letters were recorded. Data collected included global rank (top 10%: star student, chief resident potential; top 1/3: above average student; middle 1/3: solid student, average; and bottom 1/3: below average student), grade (honors, high pass, pass, low pass, fail), and specific category (knowledge, work ethic, communication, teachability, respectfulness, admits mistakes, accountable, and reliability) rank (top 1/3: above peers; middle 1/3: at level of peers; bottom 1/3: below peers). OSLOEs were excluded from review if they were submitted from EM or EM subspecialty rotations, no rotation was identified or the rotation was non-clinical or obscure (e.g., research, administrative). Recorded data were reviewed and checked for accuracy by the principal investigator prior to statistical summary. A total of 296 OSLOEs were submitted with 598 applications, with 270 meeting the eligibility criteria for review. Summary statistics were calculated using IBM SPSS Statistics, v. 23 (Armonk, NY; IBM Corp).

### Ethical Consideration

The study was reviewed and determined to be non-human subjects’ research by the Institutional Review Board.

## Results

A total of 598 applications were submitted. Of these, 296 OSLOEs were submitted from 232 (38.8%) applicants (1 OSLOE: 74.6%, 2 OSLOEs: 23.3%, 3 OSLOEs: 1.3%). Twenty-six OSLOEs were excluded due to EM or EM subspecialty rotations, no rotation identified, or non-clinical or obscure rotations, leaving 270 OSLOEs for review.  

**Figure 1:**
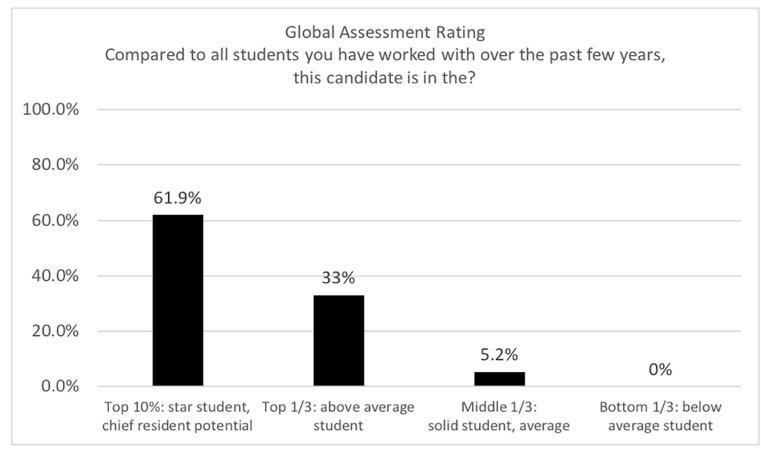
Global assessment rating

**Figure 2:**
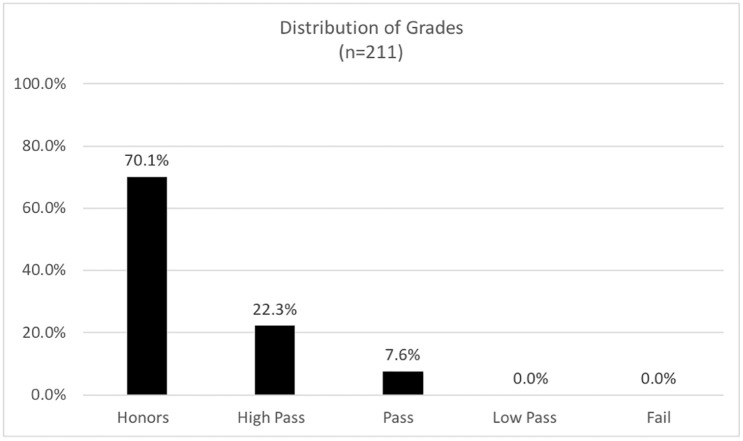
Distribution of grade

**Figure 3:**
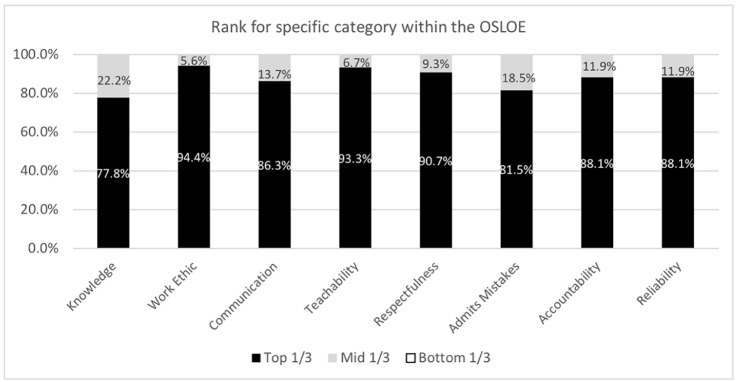
Rank for specific category within the OSLOE (Off Standardized Letters of Evaluation)

For the overall global assessment rating comparing the student “to all students you have worked with over the past few years” 95% of the letters ranked the candidate in the top 2 categories (Top 10%: star student, chief resident potential; Top 1/3: above average student), with over 60% rated in the highest category (Figure 1). There were 211 OSLOEs with grades given, with over 90% receiving the highest two grades (honors, high pass) and 0% in the bottom 2 categories of low pass or fail (Figure 2). Over 75% of the OSLOEs had rankings of top 1/3 for specific characteristics as compared to their peers and 0% ranked as bottom 1/3 (Figure 3). 

## Discussion

Given the overall ranking and grade distributions seen in our study, the utility of the objective portion of the OSLOE seems quite low. Our findings showed that the majority of submitted OSLOEs ranked applicants in the top 10 % (star student, chief resident potential) and 95% ranked students in the top two categories. The majority of students were given the highest grade (honors) and highest ranking (top 1/3) in specific categories as well. In contrast, authors reviewing rank distributions of an electronic version of the SLOE reported findings of only 18% of candidates receiving a rank of top 10% and only 55% received the highest two rankings ( 5 ). Further, only 39% received an honors grade. This earlier study also demonstrated that prior to the revision and training efforts related to the SLOE, it suffered from over-ranking of applicants. 

We suspect a contributing factor to the lack of utility regarding OSLOEs submitted to our program is the evaluators’ lack of familiarity with how to use the tool, especially as it relates to the EM residency application process. A previous study found less experienced evaluators were more likely to grade favorably, even among EM faculty who write SLOEs annually ( 12 ). Gender, duration of time the letter writer knew the applicant, inexperience and rotations at the home institution have also been shown to cause bias when evaluating candidates ( 13 - 15 ). SLOEs have been through multiple iterations, with an educational campaign and series of best practices for the people utilizing them most ( 16 ).

Selection bias may also play a role in the lack of utility of the OSLOE. Given the freedom of a student to choose the faculty at their home institution to complete the OSLOE, it would be foolish not to consider that they would select someone they believe will rank them highly. This is fundamentally different than the SLOE, in which only certain faculty / faculty groups can prepare SLOEs within EM, and students are not allowed to choose who completes the evaluation. Evaluators also may have graded higher given the situation the students were put in due to the pandemic. 

A limitation of our findings is that results are from one academic cycle at a single institution. However, the data reported was generated from various specialties from all over the country within these OSLOEs. A larger multi-institutional comparison would help to elucidate the utility of the OSLOE.

## Conclusion

In an attempt to adapt quickly to the lack of availability of in-person EM rotations due to the COVID-19 pandemic, the OSLOE was a logical alternative. However, the utility of the OSLOE in place of a traditional SLOE remains in question. Our findings provide evidence that the ranking distribution of the OSLOE may have little value in the evaluation of student performance. If the OSLOE continues to be utilized, educational efforts related to completing these letters of recommendation may be warranted.

## Authors' contribution

J.G, B.D, J.C, Contributed to data collection and manuscript review; T.K, Performed statistical analysis and manuscript review; R.O, Performed manuscript review; J.B Implemented study design, and contributed to manuscript review. All Authors contributed in drafting and revising the manuscript critically for important intellectual content. All authors have read and approved the final manuscript and agree to be accountable for all aspects of the work in ensuring that questions related to the accuracy or integrity of any part of the work are appropriately investigated and resolved.

## Conflict of Interest:

None declared.
